# CaWRKY50 Acts as a Negative Regulator in Response to *Colletotrichum scovillei* Infection in Pepper

**DOI:** 10.3390/plants12101962

**Published:** 2023-05-11

**Authors:** Yang Li, Xiao Ma, Luo-Dan Xiao, Ya-Nan Yu, Hui-Ling Yan, Zhen-Hui Gong

**Affiliations:** 1 College of Horticulture, Northwest A&F University, Yangling 712100, China; liyang1993@nwafu.edu.cn (Y.L.); mxiao26@163.com (X.M.); yyn18233281502@139.com (Y.-N.Y.); 2 Yibin Research Institute of Tea Industry, Yibin 644000, China; xldcy124@163.com; 3 Institute of Cash Crops, Hebei Academy of Agricultural and Forestry Sciences, Shijiazhuang 050051, China

**Keywords:** anthracnose, fungal pathogens, CaWRKY50, *Colletotrichum scovillei*, pepper

## Abstract

Chili anthracnose is one of the most common and destructive fungal pathogens that affects the yield and quality of pepper. Although WRKY proteins play crucial roles in pepper resistance to a variety of pathogens, the mechanism of their resistance to anthracnose is still unknown. In this study, we found that *CaWRKY50* expression was obviously induced by *Colletotrichum scovillei* infection and salicylic acid (SA) treatments. *CaWRKY50*-silencing enhanced pepper resistance to *C. scovillei*, while transient overexpression of *CaWRKY50* in pepper increased susceptibility to *C. scovillei*. We further found that overexpression of *CaWRKY50* in tomatoes significantly decreased resistance to *C. scovillei* by SA and reactive oxygen species (ROS) signaling pathways. Moreover, CaWRKY50 suppressed the expression of two SA-related genes, *CaEDS1* (*enhanced disease susceptibility 1*) and *CaSAMT1* (*salicylate carboxymethyltransferase 1*), by directly binding to the W-box motif in their promoters. Additionally, we demonstrated that CaWRKY50 interacts with CaWRKY42 and CaMIEL1 in the nucleus. Thus, our findings revealed that CaWRKY50 plays a negative role in pepper resistance to *C. scovillei* through the SA-mediated signaling pathway and the antioxidant defense system. These results provide a theoretical foundation for molecular breeding of pepper varieties resistant to anthracnose.

## 1. Introduction

Pepper (*Capsicum annuum* L.) is one of the most commercially significant vegetables in the world [1]. However, pepper cultivation is often damaged by pathogenic fungi, including chili anthracnose caused by *Colletotrichum* spp. [2]. Among these fungi, *C. scovillei* is one of the most devastating fungal diseases in pepper [3]. Currently, traditional chemical treatments are widely used to control chili anthracnose, but they are harmful to the environment and can negatively impact food safety. The breeding of resistant pepper varieties is the most effective and economical approach to controlling plant pathogens [4]. Therefore, it is important to study the mechanisms of pepper resistance to anthracnose.

Salicylic acid (SA) serves as a vital hormone for plant defense and plays a crucial role in many aspects of plant immunity. SA is involved not only in systemic acquired resistance (SAR) but also in PAMP-triggered immunity (PTI) and effector-triggered immunity (ETI) [5]. The PTI and ETI immune systems cooperate to promote downstream responses, leading to the production of ROS and plant hormones, as well as the activation of a hypersensitive response (HR) to trigger associated defense mechanisms [6]. When plants are attacked by pathogens, SA stimulates the synthesis of pathogenesis-related (PR) proteins involved in plant defense [7]. Under stress combinations, plants can enhance their levels of antioxidant enzymes to decrease ROS (particularly H_2_O_2_ and O_2_^−^) accumulation and reduce their damage by maintaining a dynamic balance [8].

Salicylate carboxymethyltransferase (SAMT) converts SA to methyl salicylate (MeSA) and plays a key role in controlling SA synthesis, affecting the defense response to pathogens in tomatoes [9,10]. *CsWRKY70* regulates citrus fruit resistance against *P. digitatum* by activating the expression of *CsSAMT* [11]. *EDS1* is required for innate immunity, and the EDS1-mediated SA-signaling pathway is important for pathogen resistance [12]. Further research has shown that EDS1 interacts with PAD4 (phytoalexin deficiency 4) and SAG101 (senescence-associated gene 101) to trigger immunity, which are essential regulators of plant defense against various pathogens [13].

WRKY transcription factors (TF), which contain a conserved WRKYGQK sequence, play important roles in pathogen-response signaling networks [14]. Previous studies have shown that many WRKY proteins are involved in SA-mediated defense signaling pathways. *WRKY38* and *WRKY62* expression in *Arabidopsis* were induced by SA and participated in SA signaling pathways [15]. While *VqWRKY31* in grapevines improves powdery mildew resistance by SA defense signaling [16]. In apples, MdWRKY17 promoted the expression of *MdDMR6* (SA degradation gene), resulting in a decreased response to *C. fructicola* inoculation [17]. In citrus, exogenous SA regulates *CsWRKY70* expression and MeSA synthesis to enhance resistance against *Penicillium digitatum* [11]. In tobacco, NbWRKY40 regulates the expression of SA-related genes in response to tomato mosaic virus resistance [18].

Numerous studies have demonstrated the critical role of WRKY transcription factors (TFs) in pepper defense responses to pathogen inoculation. *CaWRKY40* is involved in various biological processes and plays a crucial function in the response to *Ralstonia solanacearum* (RSI) infection [19,20,21]. Previous research has revealed that *CaWRKY27* [22], *CaWRKY6* [23], *CaWRKY22* [24], *CaWRKY41* [25], *CaWRKY30* [26], *CaWRKY28* [27], and *CaWRKY27b* [19] positively regulate resistance response to RSI, while *CaWRKY58* [28] and *CaWRKY40b* [29] negatively regulate it. However, the regulatory mechanism of pepper resistance to anthracnose remains unclear. Therefore, it is of great significance to explore the molecular mechanism of resistance to anthracnose in pepper.

In this study, we isolated the CaWRKY50 TF from the anthracnose-susceptible cultivar cv. R25, which was markedly induced by *C. scovillei* inoculation and SA treatments. Silencing of *CaWRKY50* increased pepper fruit resistance to *C. scovillei*, while overexpression of CaWRKY50 decreased anthracnose resistance in pepper and tomato fruits. Moreover, *CaWRKY50* specifically binds to the *CaEDS1* and *CaSAMT1* gene promoters and suppresses their expression. In the nucleus, CaWRKY50 interacts with CaWRKY42 and CaMIEL1. Our study demonstrates that CaWRKY50 negatively regulates pepper fruit resistance to *C. scovillei* through SA and ROS signaling pathways.

## 2. Results

### 2.1. Characterization of CaWRKY50

**Figure 1:**
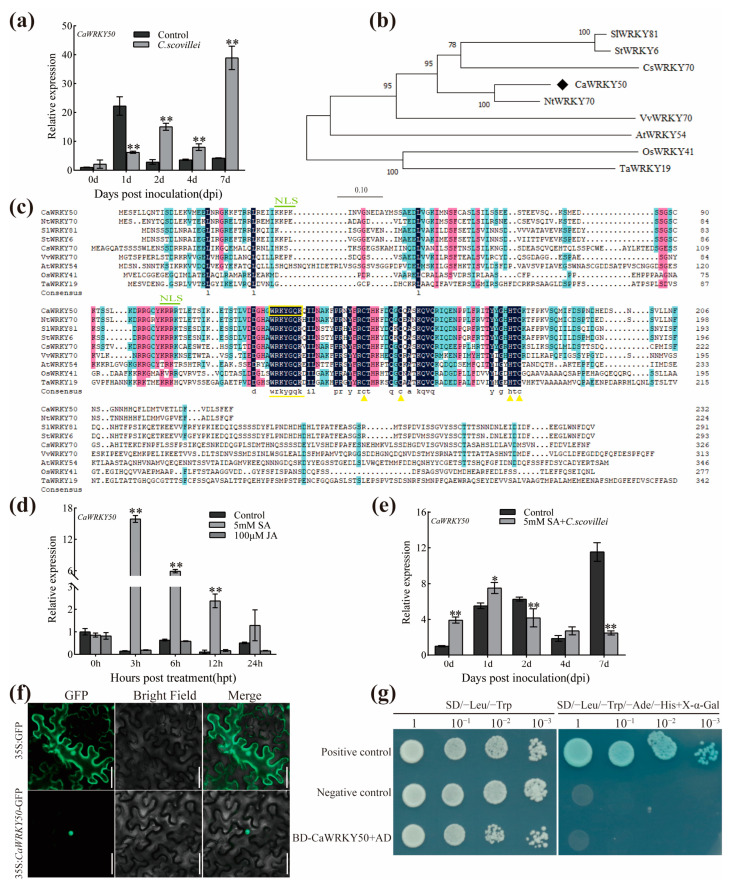
Characterization of *CaWRKY50*. (**a**) Relative transcript levels of *CaWRKY50* under *C. scovillei* inoculation in cv. R25 pepper fruits. (**b**) The phylogenetic tree of CaWRKY50 and other related WRKY proteins. The black asterisk indicates CaWRKY50. (**c**) Multiple sequence alignment of *CaWRKY50* and homologous WRKY proteins. In (**b**,**c**), the sequences are from the following proteins: NtWRKY70 (*Nicotiana tabacum*, XP_016492893.1), SlWRKY81 (*Solanum lycopersicum*, NP_001266272.1), StWRKY6 (*Solanum tuberosum*, NP_001275414.1), CsWRKY70 (*Citrus sinensis*, KAH9673391.1), VvWRKY70 (*Vitis vinifera*, XP_002275401.1), AtWRKY54 (*Arabidopsis thaliana*, AT2G40750.1), OsWRKY41 (*Oryza sativa*, XP_015638413.1), and TaWRKY19 (*Triticum aestivum*, XP_04438106). (**d**) Expression analysis of *CaWRKY50* in response to 5 mM SA and 100 μM JA treatment. (**e**) Transcript analysis of *CaWRKY50* following *C. scovillei* inoculation after exogenous 5 mM SA treatment in pepper fruits. *CaUBI3* (AY486137) was used as the internal control. Data are shown as means (±SD) from three biological replicates. Significant differences are shown by asterisks (* *p* < 0.05, ** *p* < 0.01, Student’s *t* test). (**f**) Localization of the CaWRKY50 protein in *N. benthamiana* epidermal cells (Scale bar = 50 μm). The 35S:GFP protein is used as a control. (**g**) Transcriptional activation of CaWRKY50 in the yeast. The positive control is pGBKT7-53 and pGADT7-T, and the negative control is pGBKT7-Lam and pGADT7-T.

To explore the potential function of *CaWRKY50* in response to anthracnose, we used qRT-PCR to investigate any change in *CaWRKY50* expression in the susceptible cv. R25 under *C. scovillei* infection. We found that *CaWRKY50* expression was significantly induced from 1 to 7 dpi compared to before infection, indicating that *CaWRKY50* was involved in disease resistance following *C. scovillei* infection in pepper fruits (Figure 1a). The coding sequence of *CaWRKY50* (Capana10g001548) was cloned from cv. R25 and encodes a protein consisting of 232 amino acids. Phylogenetic analysis revealed that CaWRKY50 was highly identical to NtWRKY70 (XP_016492893.1) and homologous to AtWRKY54 (AT2G40750.1), SlWRKY81 (NP_001266272.1), StWRKY6 (NP_001275414.1), VvWRKY70 (XP_002275401.1), CsWRKY70 (KAH9673391.1), OsWRKY41 (XP_015638413.1), and TaWRKY19 (XP_04438106) based on BLAST analysis in the NCBI database (Figure 1b). The CaWRKY50 protein contained one conserved WRKYGQK domain, a C2HC zinc finger, and was clustered in subgroup III members (Figure 1c).

To understand the effect of SA and jasmonic acid (JA) on pepper fruit resistance against *C. scovillei*, we analyzed the expression patterns of the *CaWRKY50* gene after treatment with 5 mM SA and 100 μM JA. The results show that *CaWRKY50* was strongly induced at 3–12 hpt with 5 mM SA but did not respond to 100 μM JA (Figure 1d). We also analyzed the transcript levels of *CaWRKY50* following *C. scovillei* infection after exogenous 5 mM SA treatment using qRT-PCR. The results indicate that *CaWRKY50* expression was significantly decreased compared to the control at 2 and 7 dpi (Figure 1e). These findings imply that the response of *CaWRKY50* to *C. scovillei* may be related to SA signaling pathways.

Sequence analysis using the PSORT online program revealed that the predicted CaWRKY50 protein contains two putative nuclear localization signals (Figure 1c). To confirm this speculation, the recombinant CaWRKY50-GFP protein was transiently expressed in tobacco epidermal cells. The results show that the CaWRKY50-GFP protein only appeared in the nucleus, while the empty GFP protein was present throughout the cell (Figure 1f). To analyze the transcriptional activity of CaWRKY50, we used the yeast two-hybrid (Y2H) system. The positive control grew well and turned blue in the SD/-Leu/-Trp/-Ade/-His medium with X-α-gal (20 μg mL^−1^), while pGBKT7-CaWRKY50 and the negative control did not express X-α-gal activity (Figure 1g). These results suggest that CaWRKY50 has no transcriptional activity in yeast cells.

### 2.2. CaWRKY50 Silencing Improves Pepper Resistance to C. scovillei

**Figure 2:**
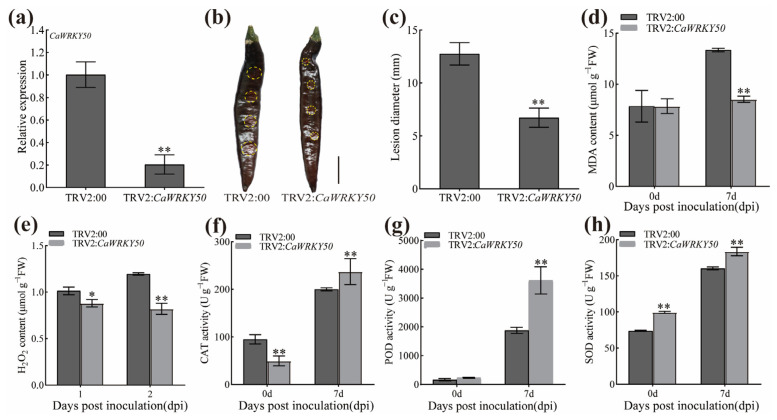
Phenotypic and physiological index analysis of *CaWRKY50*-silenced pepper fruits. (**a**) The silencing efficiency of *CaWRKY50* in pepper fruits was detected by qRT-PCR at 15 days post-infiltration. (**b**) The disease symptoms of *CaWRKY50*-silenced fruits infected with *C. scovillei* at 7 dpi (bar = 20 mm). The lesion regions are denoted by the yellow dotted line. (**c**) Lesion diameters were measured at 7 dpi. (**d**) MDA content. (**e**) H_2_O_2_ content. (**f**) CAT activity. (**g**) POD activity. (**h**) SOD activity. *CaUBI3* (AY486137) was used as the internal control. Data are shown as means (±SD) from three biological replicates. Significant differences are shown by asterisks (* *p* < 0.05, ** *p* < 0.01, Student’s *t* test).

To investigate the function of *CaWRKY50* in anthracnose resistance, we downregulated its expression on detached pepper fruits (cv. R25) using virus-induced gene silencing (VIGS) methods. *CaWRKY50* expression was significantly lower in silenced pepper fruits compared to control fruits at 15 days post-infiltration, which indicates that *CaWRKY50* were successfully silenced (Figure 2a). The phenotypic symptoms and lesion diameters of *CaWRKY50*-silenced fruits were considerably smaller in comparison to control fruits at 7 dpi (Figure 2b,c). Furthermore, the contents of malondialdehyde (MDA) and H_2_O_2_ in *CaWRKY50*-silenced fruits were remarkably lower than those in control fruits at 7 dpi (Figure 2d,e). Conversely, the catalase (CAT), peroxidase (POD), and superoxide dismutase (SOD) activities of *CaWRKY50*-silenced fruits were significantly higher at 7 dpi (Figure 2f–h).

**Figure 3:**
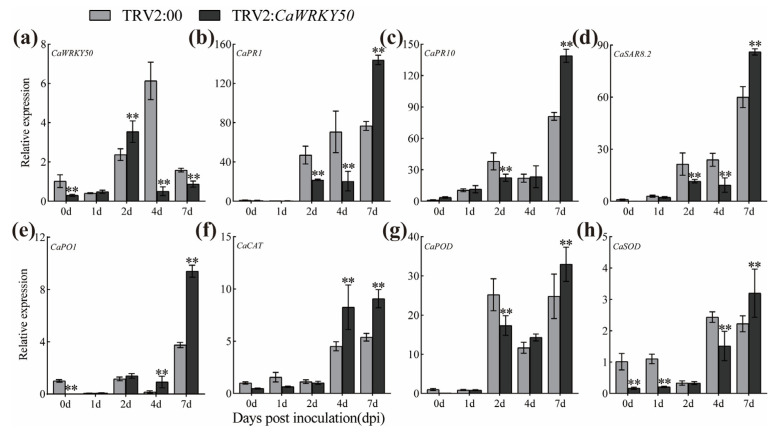
Expression profiles of defense-related genes in *CaWRKY50*-silenced fruits. (**a**) The expression level of *CaWRKY50* in *CaWRKY50*-silenced and control fruits after *C. scovillei* infection. (**b**–**e**) The transcript patterns of *CaPR1*, *CaPR10*, *CaSAR8.2*, and *CaPO1* (SA-related genes) in control and *CaWRKY50*-silenced fruits under *C. scovillei* infection. (**f**–**h**) The transcript patterns of *CaCAT*, *CaPOD*, and *CaSOD* (ROS-related genes) in controls and *CaWRKY50*-silenced fruits under *C. scovillei* infection. *CaUBI3* (AY486137) is used as the internal control. Data are shown as means (±SD) from three biological replicates. Significant differences are shown by asterisks (** *p* < 0.01, Student’s *t* test).

Furthermore, the expression of *CaWRKY50* was markedly lower in *CaWRKY50*-silenced fruits compared to control fruits at 4 and 7 dpi (Figure 3a). To determine whether *CaWRKY50* affects the expression of SA and ROS defense-related genes in pepper, qRT-PCR was used to evaluate the expression levels of these genes at various days after *C. scovillei* infection. The transcript levels of *CaPR1*, *CaPR10*, *CaSAR8.2*, and *CaPO1* (SA-related genes) were significantly increased in *CaWRKY50*-silenced fruits relative to control fruits at 7 dpi (Figure 3b–e). Similarly, the transcript patterns of *CaCAT*, *CaPOD*, and *CaSOD* (ROS-related genes) were consistent with the SA-related genes (Figure 3f–h). Overall, these results indicate that silencing *CaWRKY50* improves the resistance of pepper fruits to *C. scovillei*.

### 2.3. Transient CaWRKY50 Overexpression Enhances Susceptibility to C. scovillei in Pepper

**Figure 4:**
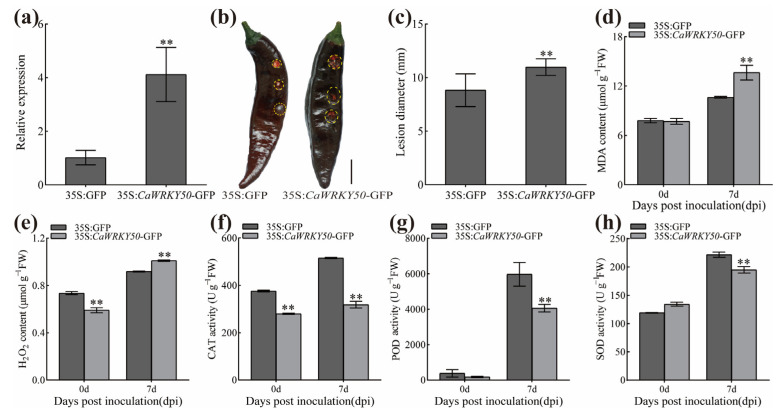
Phenotypic and physiological index analysis of *CaWRKY50* transient overexpression in pepper fruits. (**a**) The success of transient overexpression of *CaWRKY50* in pepper fruits was detected by qRT-PCR at 3 days post-infiltration. (**b**) The disease symptoms of *CaWRKY50* transient overexpression fruits infected with *C. scovillei* at 7 dpi (bar = 20 mm). The lesion regions are denoted by the yellow dotted line. (**c**) Lesion diameters were measured at 7 dpi. (**d**) MDA content. (**e**) H_2_O_2_ content. (**f**) CAT activity. (**g**) POD activity. (**h**) SOD activity. *CaUBI3* (AY486137) was used as the internal control. Data are shown as means (±SD) from three biological replicates. Significant differences are shown by asterisks (** *p* < 0.01, Student’s *t* test).

To further explore the role of *CaWRKY50* in disease resistance to anthracnose, we performed transient overexpression of *CaWRKY50* in detached pepper fruits (cv. R25) and infected them with *C. scovillei*. *CaWRKY50* was successfully overexpressed in the pepper fruits, as confirmed by qRT-PCR analysis at 3 days post-infiltration (Figure 4a). The disease symptoms and lesion diameters of *CaWRKY50*-overexpressing fruits were markedly larger than those of control fruits at 7 dpi (Figure 4b,c). Additionally, MDA and H_2_O_2_ levels in *CaWRKY50*-overexpressing fruits were dramatically increased compared to the control fruits at 7 dpi (Figure 4d,e). Furthermore, the CAT, POD, and SOD activities of *CaWRKY50*-overexpressing fruits were significantly lower than those of control fruits at 7 dpi (Figure 4f–h).

**Figure 5:**
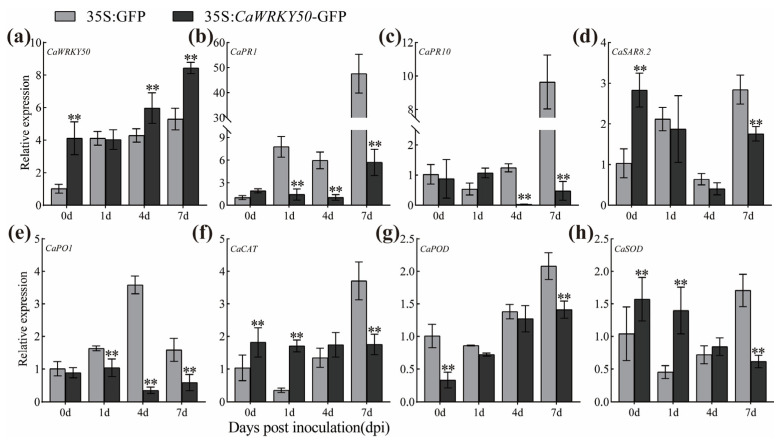
Expression profiles of defense-related genes in *CaWRKY50* transient overexpression. (**a**) The expression level of *CaWRKY50* in transient overexpression and control fruits after *C. scovillei* infection. (**b**–**e**) The transcript patterns of *CaPR1*, *CaPR10*, *CaSAR8.2*, and *CaPO1* (SA-related genes) in control and *CaWRKY50* transient overexpression fruits under *C. scovillei* infection. (**f**–**h**) The transcript patterns of *CaCAT*, *CaPOD*, and *CaSOD* (ROS-related genes) in the control and *CaWRKY50* transient overexpression fruits under *C. scovillei* infection. *CaUBI3* (AY486137) is used as the internal control. Data are shown as means (±SD) from three biological replicates. Significant differences are shown by asterisks (** *p* < 0.01, Student’s *t* test).

Additionally, *CaWRKY50* expression was obviously increased in *CaWRKY50*-overexpressing fruits compared to control fruits at 4 and 7 dpi (Figure 5a). To understand whether *CaWRKY50* regulates the expression of SA and ROS-related defense genes, we analyzed the transcript levels of these genes in *CaWRKY50*-overexpressing fruits as well as control fruits at various days after *C. scovillei* infection. The transcript levels of *CaPR1*, *CaPR10*, *CaSAR8.2*, and *CaPO1* (SA-related genes) were downregulated in the *CaWRKY50*-overexpressing fruits relative to the control at 7 dpi (Figure 5b–e). Similarly, the expression patterns of *CaCAT*, *CaPOD* and *CaSOD* (ROS-related genes) were consistent with the SA-related genes under *C. scovillei* infection at 7 dpi (Figure 5f–h). Taken together, our data show that *CaWRKY50* negatively regulates pepper fruit resistance to *C. scovillei*.

### 2.4. Overexpression of CaWRKY50 in Tomato Decreases Resistance to C. scovillei

**Figure 6:**
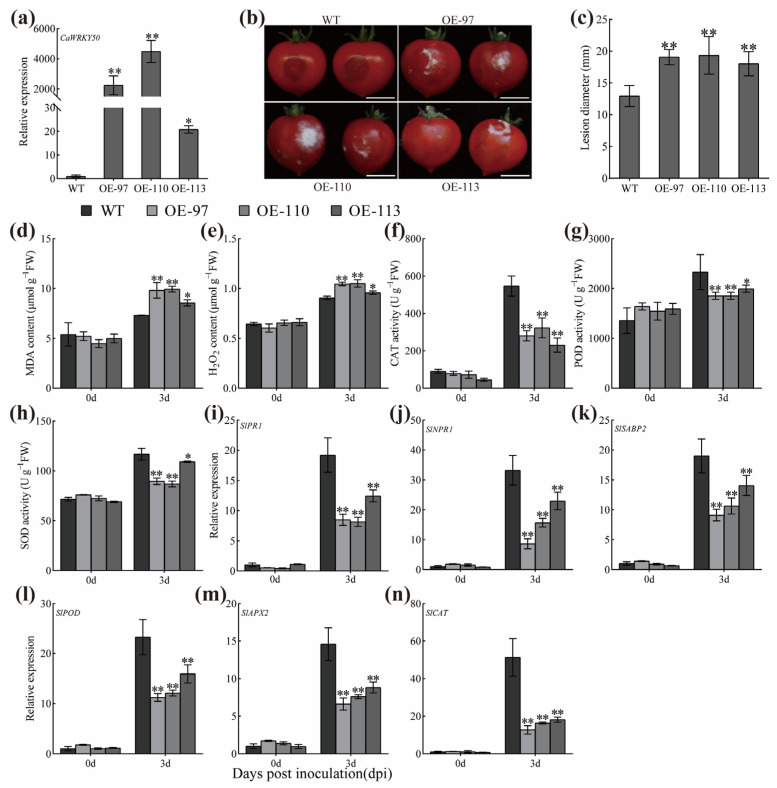
Heterologous overexpression of *CaWRKY50* in tomatoes. (**a**) qRT-PCR analysis of *CaWRKY50* in transgenic tomatoes. (**b**) The symptoms of *CaWRKY50* transgenic lines and control fruits infected with *C. scovillei* at 3 days post-inoculation (dpi) (bar = 10 mm). (**c**) The lesion diameter of *CaWRKY50* transgenic lines and control fruits were measured at 3 dpi. (**d**) MDA content. (**e**) H_2_O_2_ content. (**f**) CAT activity. (**g**) POD activity. (**h**) SOD activity. (**i**–**k**) qRT-PCR analysis of SA-related defense genes (*SlPR1*, *SlNPR1*, and *SlSABP2*) in OE and control fruits at 0 and 3 dpi. (**l**–**n**) qRT-PCR analysis of ROS-related genes (*SlPOD*, *SlAPX2*, and *SlCAT*) at 0 and 3 dpi. *SlACTIN* (NM_001308447.1) was used as the internal control. Data are shown as means (±SD) from three biological replicates. Significant differences are shown by asterisks (* *p* < 0.05, ** *p* < 0.01, Student’s *t* test).

To better understand the function of *CaWRKY50* in anthracnose resistance, *CaWRKY50* was stably expressed in tomato by *Agrobacterium*-mediated genetic transformation. Three transgenic T_2_ lines (OE-97, OE-110, and OE-113) with high levels of *CaWRKY50* transcript (Figure 6a) were infected with *C. scovillei* to assess the role of *CaWRKY50* in anthracnose resistance. At 3 dpi, the transgenic fruits exhibited greater lesion diameters than WT fruits (Figure 6b,c). MDA and H_2_O_2_ content were evaluated in OE and WT fruits, and compared to WT fruits, MDA and H_2_O_2_ levels were greatly increased in OE fruits at 3 dpi (Figure 6d,e). Moreover, the activities of CAT, POD, and SOD were obviously lower in OE fruits than WT fruits at 3 dpi (Figure 6f–h).

Additionally, we analyzed the expression levels of SA and ROS related defense genes in both *CaWRKY50*-overexpressing and control fruits. The transcript patterns of SA-related genes *SlPR1*, *SlNPR1*, and *SlSABP2* were markedly downregulated in CaWKRY50 OE fruits compared to WT fruits at 3 dpi (Figure 6i–k). Consistently, the transcript patterns of the ROS-related genes *SlPOD*, *SlAPX2*, and *SlCAT* were also significantly downregulated in OE fruits relative to WT fruits at 3 dpi (Figure 6l–n). In conclusion, our studies demonstrate that *CaWRKY50* has a negative regulatory effect on tomato fruit resistance to *C. scovillei*.

### 2.5. CaWRKY50 Specifically Binds the Promoter of CaEDS1 and CaSAMT1

**Figure 7:**
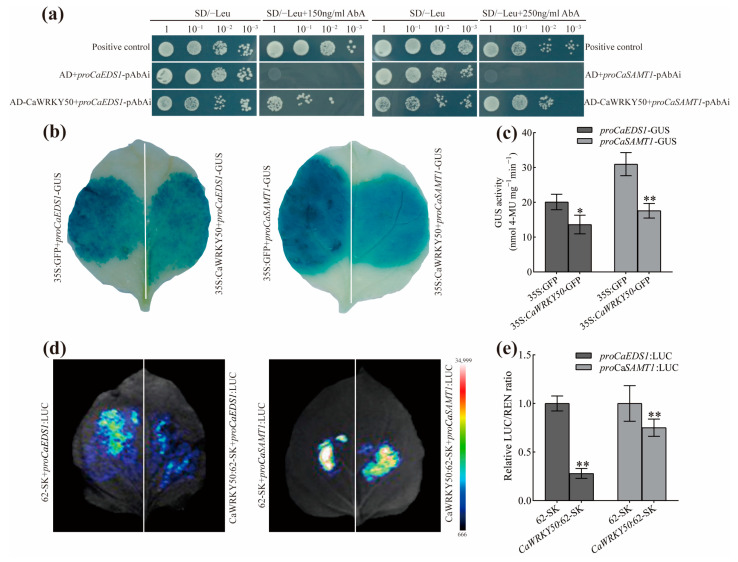
CaWRKY50 directly binds to *CaEDS1* and *CaSAMT1* promoters and negatively regulates their transcriptional activity. (**a**) Yeast one-hybrid (Y1H) analysis of CaWRKY50 directly binds to the promoter of *CaEDS1* and *CaSAMT1*. The transformed yeast cells were screened on SD/-Leu medium with 150 and 250 ng mL^−1^ AbA. The positive control is the co-transformation of pGADT7 and pAbAi-p53, and the negative control is the co-transformation of pGADT7 and pAbAi-bait. (**b**,**c**) GUS analysis of the influence of CaWRKY50 on the activity of the *proCaEDS1/proCaSAMT1*. (**b**) Histochemical GUS staining. (**c**) Quantification of GUS activity. (**d**,**e**) The effect of CaWRKY50 on the activity of the *CaEDS1* and *CaSAMT1* promoters by dual-luciferase assay in tobacco (*Nicotiana benthamiana*) leaves. The LUC to REN ratio was assessed as transcriptional activity. The empty vector 62-SK was used as a control. Data are shown as means (±SD) from three biological replicates. Significant differences are shown by asterisks (* *p* < 0.05, ** *p* < 0.01, Student’s *t* test).

To identify which genes are directly regulated by CaWRKY50 for anthracnose resistance in pepper, we looked for two SA-related defense genes (*CaEDS1* and *CaSAMT1*), whose promoters contain W-box elements (TTGACC/T). Subsequently, we performed yeast one-hybrid (Y1H) assays to confirm whether CaWRKY50 binds to the promoter of *CaEDS1* and *CaSAMT1*. We transformed CaWRKY50-pGADT7 into the Y1H strain by integrating the *proCaEDS1*/*proCaSAMT1*-pAbAi vector, which produced yeast cells that grew well on SD/-Leu medium with 150 and 250 ng ml^−1^ AbA. In contrast, the negative control failed to grow (Figure 7a). The Y1H assay confirmed that CaWRKY50 specifically binds to the promoters of *CaEDS1* and *CaSAMT1*.

To investigate the effect of CaWRKY50 on the promoters of *CaEDS1* and *CaSAMT1*, we performed GUS activity and dual-luciferase assays in tobacco leaves. The staining intensity and activity of GUS were significantly decreased compared to the control when CaWRKY50-pGADT7 was co-expressed with the *proCaEDS1*/*proCaSAMT1*-GUS vector in tobacco leaves (Figure 7b,c). Furthermore, we observed that the luciferase activity was significantly inhibited compared to control when CaWRKY50:62-SK was co-expressed with the *proCaEDS1*/*proCaSAMT1*-LUC vector in tobacco leaves (Figure 7d,e). Taken together, these results indicate that CaWRKY50 specifically binds to the promoters of *CaEDS1* and *CaSAMT1*, suppressing their expression in response to *C. scovillei*.

### 2.6. CaWRKY50 Interacts with CaWRKY42 and CaMIEL1

**Figure 8:**
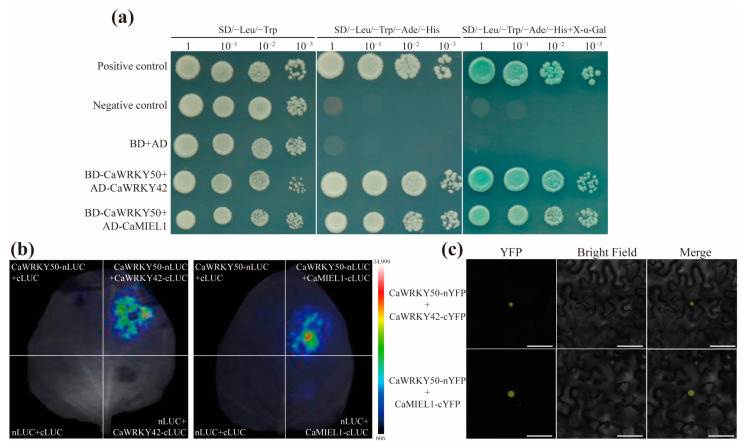
The interaction of CaWRKY50 and CaWRKY42/CaMIEL1. (**a**) Yeast two-hybrid (Y2H) assay analysis of the interaction of CaWRKY50 and CaWRKY42/CaMIEL1. Different plasmid combinations were co-transformed into Y2H Gold. The positive controls are pGBKT7-53 and pGADT7-T, and the negative controls are pGBKT7-Lam and pGADT7-T. (**b**) Luciferase complementation imaging (LCI) assays. CaWRKY50-nLUC and CaWRKY42/CaMIEL1-cLUC were transiently co-expressed in tobacco leaves. Luciferase signals were imaged at 48 hpi. (**c**) Bimolecular fluorescence complementation (BiFC) assays. CaWRKY50-nYFP and CaWRKY42/CaMIEL1-cYFP were transiently co-expressed in tobacco leaves. YFP signals were observed at 48 hpi. Scale bars = 50 μm.

In order to identify the interacting partner of CaWRKY50 in the regulation of pepper defense responses, we screened a pepper cDNA library by Y2H assay and isolated CaWRKY42 (Capana08g001044) and CaMIEL1 (XM_016719768) as CaWRKY50-interacting proteins. CaMIEL1 encodes a RING E3 ubiquitin-protein ligase. Subsequently, we carried out a Y2H assay to confirm whether CaWRKY50 interacted with CaWRKY42/CaMIEL1. The yeast cells co-transformed with CaWRKY50-pGBKT7 and CaWRKY42/CaMIEL1-pGADT7 plasmid combinations were viable on SD/-Leu/-Trp/-Ade/-His medium and turned blue in X-α-gal (20 μg mL^−1^) (Figure 8a). This confirmed that CaWRKY50 strongly interacted with CaWRKY42 and CaMIEL1.

Moreover, we performed luciferase complementation imaging (LCI) and bimolecular fluorescence complementation (BiFC) assays to confirm the interactions in living plant cells. In the LCl assay, we transiently co-expressed CaWRKY50-nLUC and CaWRKY42/CaMIEL1-cLUC in tobacco leaves and found luminescence signals in the co-expressed CaWRKY50-nLUC and CaWRKY42/CaMIEL1-cLUC regions but not in controls (Figure 8b). In the BiFC assay, we transiently co-expressed CaWRKY50-nYFP and CaWRKY42/CaMIEL1-cYFP in tobacco leaves and observed the interaction between CaWRKY50 and CaWRKY42/CaMIEL1 generating YFP fluorescence in the nucleus (Figure 8c). In summary, our data demonstrate that CaWRKY50 interacts with CaWRKY42/CaMIEL1 in the nucleus.

## 3. Discussion

WRKY proteins play a critical role in plant resistance to various pathogens. According to the *Zunla-1* genome sequence, a total of 62 WRKY genes have been identified in pepper [30]. In our study, we selected *CaWRKY50* as a candidate gene based on previous RNA-seq and VIGS analysis. Sequence analysis indicated that CaWRKY50 is highly homologous with NtWRKY70 in tobacco, AtWRKY54 in *Arabidopsis*, and CsWRKY70 in citrus. These proteins belong to subgroup III members (Figure 1b,c), for which *AtWRKY54* plays a positive regulatory role in pathogen infection and a negative regulatory role in leaf senescence [31,32]. *CsWRKY70* actively participates in SA-mediated disease resistance by regulating the transcription of the *CsSAMT* genes [11]. Thus, it was speculated that *CaWRKY50* might also be involved in plant disease resistance. However, the underlying molecular mechanism of *CaWRKY50* in pepper anthracnose resistance remains unknown, and further investigation is needed to understand its function.

In this study, a transactivation assay was used to investigate CaWRKY50’s role in transcriptional activity, and it was found that it did not possess transcriptional activity in yeast cells (Figure 1g). Similar findings have been reported for grapevine VvWRKY8 [33], maize ZmWRKY17 [34], soybean GmWRKY13, GmWRKY27, GmWRKY40, GmWRKY54 [35], and *Brachypodium distachyon* BdWRKY36 [36]. This phenomenon could be explained by the fact that the activation of these proteins may be dependent on posttranslational modifications or require activation by unknown upstream proteins [37].

In this study, the transcript levels of *CaWRKY50* were markedly induced by *C. scovillei* and SA treatments, as shown in Figure 1a,d. By performing VIGS, we found that *CaWRKY50*-silencing enhanced pepper resistance to *C. scovillei* inoculation (Figure 2 and Figure 3). Also, *CaWRKY50* overexpression resulted in enhanced susceptibility to infection with *C. scovillei* in pepper and tomato fruits (Figure 4, Figure 5 and Figure 6). These results suggest that *CaWRKY50* acts as a negative regulator of pepper resistance to *C. scovillei*.

Recently, studies have revealed that plants can actively generate ROS, which can affect a variety of physiological processes, including biotic and abiotic stress as well as pathogen defense [8]. To counteract ROS damage, plants possess a comprehensive antioxidative system that reduces ROS damage and maintains cell homeostasis [38]. Malondialdehyde (MDA) is the main product of membrane lipid peroxidation and is commonly used as a marker for oxidative stress in plants [39]. For instance, overexpression of *CaCIPK13* in pepper enhanced cold tolerance by regulating the antioxidant defense system, which increased CAT, POD, and SOD activities while decreasing MDA and H_2_O_2_ content [40]. Similarly, *CaCIPK3*-overexpression improved drought stress resistance in pepper by increasing CAT, POD, and SOD activities, while decreasing MDA and H_2_O_2_ content [41]. Hence, we measured the contents of MDA and H_2_O_2_ as well as the levels of ROS scavenging enzymes (CAT, POD, and SOD) in *CaWRKY50*-overexpressing and *CaWRKY50*-silenced fruits under *C. scovillei* infection. Our results revealed that the contents of MDA and H_2_O_2_ in *CaWRKY50*-overexpressing fruits were noticeably higher than control fruits under *C. scovillei* infection (Figure 4 and Figure 6). In contrast, the results were reversed in *CaWRKY50*-silenced fruits, as shown in Figure 2. Furthermore, the activities of CAT, POD, and SOD in *CaWRKY50*-overexpressing fruits were significantly lower than control fruits under *C. scovillei* infection (Figure 4 and Figure 6), which was contrary to *CaWRKY50*-silenced fruits (Figure 2). To corroborate these findings, we measured the expression of ROS-scavenging genes (*CaCAT*/*SlCAT*, *CaPOD*/*SlPOD*, *CaSOD*, and *SlAPX2*) using qRT-PCR and found that the results were consistent with ROS-scavenging enzyme activity results (Figure 3, Figure 5, and Figure 6). These findings suggest that *CaWRKY50* plays a crucial role in regulating oxidative stress caused by *C. scovillei* infection.

SA signaling is known to play a key role in regulating the defense response of plants to pathogenic infections. Previous studies have shown that SA-related defense genes can be induced by SA to modulate plant defense responses to pathogen infection. [42]. Previous results indicated that *CaSBP12*, *CaSBP08*, and *CaSBP11* negatively regulated pepper resistance to *phytophthora capsici* by inhibiting the transcript of SA-related defense genes [39,43,44]. In another study, *VaERF16*-overexpressing improved disease resistance to *B. cinerea* in *Arabidopsis thaliana* by increasing the expression of *AtPR1* and *AtNPR1* [45]. Our research revealed that the transcript levels of *CaPR1*, *CaPR10*, *CaSAR8.2*, and *CaPO1* were significantly downregulated in transient CaWRKY50-overexpression pepper fruits in comparison to control fruits at 7 dpi. (Figure 5). In contrast, these genes were dramatically upregulated in CaWRKY50-silenced pepper fruits at 7 dpi (Figure 3). Furthermore, the expression of *SlPR1*, *SlNPR1*, and *SlSABP2* in *CaWRKY50*-overexpressing tomato fruits was markedly lower than in control fruits at 7 dpi (Figure 6i–k). In summary, these results indicate that *CaWRKY50* acts as a negative regulator in response to *C. scovillei* through the SA signaling pathway.

WRKY TFs can specifically bind to W-box cis-elements (TTGACC/T) to regulate the expression of defense-associated genes. The expression of SA signaling-related genes (*TGA2* and *TGA6*) was increased by WRKY70, which directly bound their promoter elements to regulate *Verticillium dahliae* toxin infection [46]. In the absence of a pathogen, WRKY70 is directly bound to the promoter of *SARD1* to repress its expression and regulate the expression of SA-related genes [47]. In another study, MdWRKY17 specifically bound to the *MdDMR6* promoter to enhance its expression, which negatively regulated resistance to *C. fructicola* in apples [17]. In the present study, we found that CaWRKY50 specifically binds to the promoter of *CaEDS1* and *CaSAMT1* by Y1H (Figure 7a). To elucidate how CaWRKY50 regulates the expression of *CaEDS1* and *CaSAMT1*, we conducted GUS activity and dual-luciferase assays, which demonstrate that CaWRKY50 could inhibit *CaEDS1* and *CaSAMT1* transcription (Figure 7b–e). In plants, SAMT catalyzes SA to produce MeSA. CsWRKY70 specifically binds to the promoter of *CsSAMT* and activates its expression, thereby positively regulating resistance to *Penicillium digitatum* in citrus fruit [11]. *EDS1* plays an important function in plant basal defense and the SA signaling pathway [12]. PcAvh103 interacts with EDS1 to inhibit plant immunity by the EDS1-PAD4 signaling pathway [48]. These findings suggest that CaWRKY50 can suppress the expression of *CaEDS1* and *CaSAMT1* to reduce disease resistance.

WRKY proteins can interact with other proteins to regulate plant immunity [14]. For example, WRKY33 interacts with WRKY12 to increase hypoxia tolerance in *Arabidopsis* [49]. JrWRKY21 was shown to improve walnut resistance to *C. gloeosporioides* by interacting with JrPTI5L (a PR protein) [50]. SlWRKY31 interacts with SlVQ15 to regulate the resistance to *B. cinerea* in tomatoes [51]. It is plausible that the CaWRKY50 protein also interacts with other defense proteins in response to various pathogen infections. In this study, we determined the interaction between CaWRKY50 with CaWRKY42/CaMIEL1 through Y2H, LCI, and BiFC assays (Figure 8). A previous study showed that *CaWRKY41 (CA08g08240)*, which is the same gene as *CaWRKY42 (Capana08g001044)* in our study, could positively regulate pepper resistance to *R. solanacearum* inoculation [25]. CaMIEL1, an E3 ubiquitin ligase, inhibited tolerance to bacterial inoculation in *Arabidopsis* by degrading MYB30 [52]. These results suggest that CaWRKY50 may regulate resistance to *C. scovillei* by interacting with CaWRKY42 and CaMIEL1.

**Figure 9:**
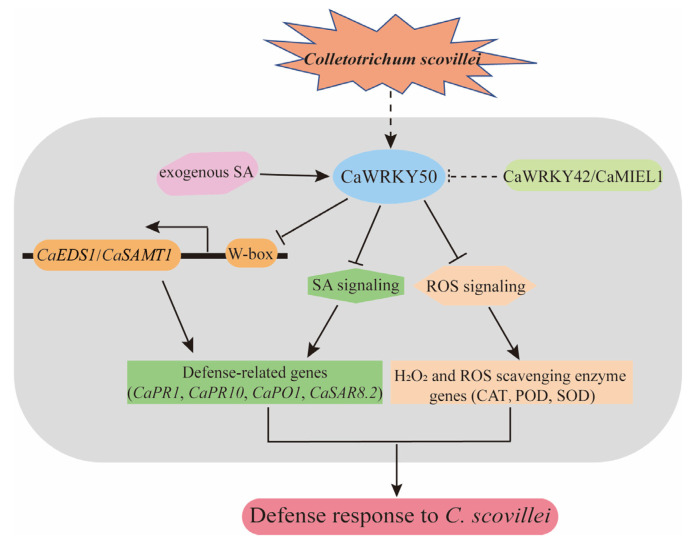
A proposed model for *CaWRKY50* in pepper under *C. scovillei* infection. *CaWRKY50* was induced under exogenous SA and *C. scovillei* stress. CaWRKY50 serves as a negative regulator in response to *C. scovillei* by regulating SA and ROS signaling pathways. CaWRKY50 directly binds to *CaEDS1* and *CaSAMT1* promoters and suppresses their expression. CaWRKY50 interacts with CaWRKY42 and CaMIEL1. Arrows indicate positive regulation, while T-bars indicate negative regulation. A solid line shows direct regulation, and dotted lines show potential regulation.

In conclusion, using overexpression and silenced techniques, we demonstrated that CaWRKY50 serves as a negative regulator in response to *C. scovillei* through the SA and ROS signaling pathways. CaWRKY50 directly binds to the promoters of *CaEDS1* and *CaSAMT1* to suppress their expression. Furthermore, CaWRKY50 participates in disease resistance by interacting with CaWRKY42 and CaMIEL1 (Figure 9). Our findings shed light on the mechanisms by which *WRKY50* functions in response to anthracnose resistance.

## 4. Materials and Methods

### 4.1. Plant Materials, Pathogen Inoculation, and Phytohormone Treatment

Pepper (*Capsicum annuum*, cv. R25), which was used for VIGS and transient overexpression assays, was cultivated in a solar greenhouse at the horticultural farm of Northwest A&F University, Shaanxi, China. The tobacco (*Nicotiana benthamiana*) was used for transient transformation. The tomato (*Solanum lycopersicum*, cv. Micro-Tom) was used for stable genetic transformation. Tomato and tobacco seedlings were grown in growth chambers at 24/18 °C under a 16/8 h photoperiod day/night cycle.

*C. scovillei* (SXBJ23) were cultured on potato dextrose agar (PDA) medium at 28 °C for 7 days. For pathogen inoculation assays, mature fruits were inoculated with *C. scovillei* using the previously described method [53]. In brief, the surfaces of mature fruits were injected with 2 μL of suspension (5 × 10^5^ spores mL^−1^) using the microinjection method [54]. The inoculated fruits were stored at 28 °C for 7 days. Measurements of lesion diameter (LD) were performed by the crossing method as previously described [55]. Pepper fruit samples were collected at 0, 1, 2, 4, and 7 days post incubation (dpi). Tomato fruit samples were collected at 0 and 3 dpi. Three biological replicates were performed for each treatment.

For the study of *CaWRKY50* transcript levels in response to SA and JA, cv. R25 mature fruits were soaked with 5 mM SA and 100 μM MeJA for 1 h [22]. Control fruits were soaked in sterile water. The pepper pericarps were collected after 0, 3, 6, 12, and 24 h post treatment (hpt) and then inoculated with 2 μL of *C. scovillei* after 24 hpt. Pepper fruits were stored at 28 °C for 7 days.

### 4.2. RNA Extraction and qRT-PCR Analysis

The total RNA was extracted by the RNAprep pure plant kit (TSP411, Tsingke, Beijing, China) according to the manufacturer’s protocol. The first-strand cDNA was synthesized using ToloScript RT EasyMix (22106, Tolobio, Shanghai, China). qRT-PCR analysis was performed using the SYBR Green Master qPCR Mix (TSE201, Tsingke, Beijing, China). *CaUBI3* (AY486137) and *SlACTIN* (NM_001308447.1) were used as reference genes for pepper and tomato to normalize transcript levels according to the 2^−ΔΔCt^ method. Each qRT-PCR experiment consisted of three biological replicates. The qRT-PCR primers listed in Supplemental Table S1.

### 4.3. Phylogenetic Analysis and Subcellular Localization

To analyze the relationship between CaWRKY50 and its homologs, the WRKY TFs, the protein sequence alignment was carried out by the DNAMAN software. The construction of the phylogenetic tree was performed using MEGA X software according to neighbor-joining (NJ).

The PSORT online program (https://www.genscript.com/psort.html (accessed on 26 April 2023)) was used to predict the subcellular localization of the CaWRKY50 protein. To confirm the subcellular localization, the coding sequence of CaWRKY50 without the stop codon was inserted into pVBG2307-GFP vectors. The recombinant construct (pVBG2307-CaWRKY50-GFP) was transformed into *Agrobacterium tumefaciens* strain GV3101 and then transiently infiltrated into tobacco (*Nicotiana benthamiana*) leaves. The empty vector serves as a negative control. The GFP fluorescence signals were detected using a BX63 Olympus (Tokyo, Japan) after 2–3 days. Fluorescence was evaluated using at least three independent replicates.

### 4.4. Transcriptional Activity Assays in Yeast

The coding sequence of CaWRKY50 was amplified and cloned into the pGBKT7 vector to investigate the transcriptional activity. Co-transformation of the CaWRKY50-pGBKT7 recombinant vector and the pGADT7-T empty vector into Y2H yeast cells according to the manufacturer’s procedures (PT1183, Shaanxi Pyeast Bio.CO.LTD, Shaanxi, China). Transformants were cultured on SD/-Leu/-Trp/-Ade/-His medium containing X-α-gal (20 μg/mL) at 30 °C for 3 days.

### 4.5. Virus-Induced Gene Silencing (VIGS)

For the VIGS assays, *CaWRKY50* silencing was performed following a previously reported procedure [56]. To generate the *CaWRKY50*-silenced vector, a 300 bp fragment of *CaWRKY50* was obtained using the VIGS Tool (https://vigs.solgenomics.net/ (accessed on 4 December 2019)). The *Agrobacterium tumefaciens* strain GV3101 cells containing TRV2:00 and TRV2:*CaWRKY50* were combined in a 1:1 ratio with TRV1 and then infiltrated into detached mature pepper fruits of cv. R25. The treated pepper fruits were placed in growth chambers at 24/18 °C under a 16/8 h photoperiod day/night cycle. After 15 days of infiltration, the transcript levels of *CaWRKY50* were measured by qRT-PCR, and then the fruits were injected with 2 μL *C. scovillei*. Pepper fruit samples were collected at 0, 1, 2, 4, and 7 dpi, and the lesion diameters were measured at 7 dpi. Three biological replicates were performed for each treatment. The experiment was repeated three times with similar results.

### 4.6. Transient and Transgenic Overexpression

We carried out transient overexpression assays by *A. tumefaciens*-mediated infiltration [57]. The fusion protein pVBG2307-CaWRKY50-GFP was infiltrated into detached mature pepper fruits of cv. R25. The expression level of *CaWRKY50* was detected at 3 days post-infiltration by qRT-PCR, and the fruits were subsequently inoculated with 2 μL *C. scovillei*. Pepper fruit samples were collected at 0, 1, 4, and 7 dpi, and the lesion diameters were measured at 7 dpi. Three biological replicates were performed for each treatment. The experiment was repeated three times with similar results.

The transgenic overexpression assays were performed as previously reported methods, with slight modifications [58]. Transcript levels in transgenic plants were evaluated by qRT-PCR. T_2_ transgenic lines (OE-97, OE-110, and OE-113) were employed for further investigation. The mature red fruits were inoculated with 2 μL of *C. scovillei* at 28 °C for 3 days. Fruit samples were collected at 0 and 3 dpi, and the lesion diameters were measured at 3 dpi. Three biological replicates were performed for each treatment.

### 4.7. Measurement of Physiological Indicators

The content of malondialdehyde (MDA) was detected according to the previously described method [40]. The catalase (CAT), peroxidase (POD), and superoxide dismutase (SOD) activities as well as H_2_O_2_ contents were quantified using the corresponding assay kits (Sangon Biotech, Shanghai, China) in accordance with the manufacturer’s procedures. Three biological replicates were performed for each determination. The experiment was repeated three times with similar results.

### 4.8. Protein Interaction Assays

The coding sequence of CaWRKY50 was cloned into the pGBKT7 vector as the bait, and the coding sequences of CaWRKY42 and CaMIEL1 were cloned into the pGADT7 vector as the prey. Co-transformation of the bait and prey vectors into Y2H yeast cells according to the manufacturer’s procedures (PT1183, Shaanxi Pyeast Bio.CO.LTD, Shaanxi, China). The protein interactions were screened using SD/-Leu/-Trp/-Ade/-His medium containing X-α-gal (20 μg/mL).

For the luciferase complementation imaging (LCI) assays, the coding sequences of CaWRKY42 and CaMIEL1 were cloned into the pCAMBIA-cLUC vector, and the coding sequence of CaWRKY50 was cloned into the pCAMBIA-nLUC vector. *Agrobacterium tumefaciens* GV3101 harboring 35S:CaWRKY50-nLUC and 35S:CaWRKY42/CaMIEL1-cLUC were co-infiltrated in *N. benthamiana* leaves. After 2 days of infiltration, 1 mM Beetle luciferin (E1601, Promega, Madison, WI, USA) was sprayed onto the inoculation leaves, and the luciferase images were captured (Lumazone Pylon 2048B, Princeton, NJ, USA). The LCI assays were carried out as previously reported [59].

For the bimolecular fluorescence complementation (BiFC) assays, the recombinant constructs CaWRKY50-pSPYNE and CaWRKY42/CaMIEL1-pSPYCE were transformed into *A. tumefaciens* strain GV3101 and co-infiltrated in tobacco leaves. An automated fluorescence microscope (BX63, Olympus, Japan) was used to image YFP signals 2 days after infiltration. The BiFC assays were carried out as previously reported [60].

### 4.9. Yeast One-Hybrid (Y1H) Assay

The coding sequence of CaWRKY50 was cloned into the pGADT7 vector, and the *CaEDS1* (799 bp) and *CaSAMT1* (521 bp) promoter regions containing W-box elements (TTGACT/C) were cloned into the pAbAi vector as a bait vector. The restriction enzyme *BstB1* was used to linearize the bait vectors before inserting them into Y1H Gold yeast cells. Then, these yeast cells were grown at 30 °C for 3 days on SD/-Leu medium with 150 and 250 ng mL^−1^ of Aureobasidin A (AbA).

### 4.10. GUS Activity Analysis and Dual Luciferase Reporter Assay

For the GUS activity analysis, the *CaEDS1* and *CaSAMT1* promoter regions were amplified from the genomic DNA of cv. R25 and inserted into the pCAMBIA1381-GUS vector as reporters. The effector plasmid, pVBG2307-CaWRKY50-GFP, was identical to that used in the previous subcellular localization assay. The recombinant constructs were transformed into *A. tumefaciens* strain GV3101 and co-infiltrated in tobacco leaves for 48 h. The GUS staining and enzyme activity detection methods were carried out as previously described [61].

For the dual luciferase reporter assays, the *CaEDS1* and *CaSAMT1* promoter regions were amplified from the genomic DNA of cv. R25 and inserted into the pGreenII-0800-LUC vector to generate a reporter construct. The coding sequence of CaWRKY50 was cloned into the pGreenII 62-SK vector to create an effector construct. The recombinant constructs were transformed into *A. tumefaciens* strain GV3101 (pSoup) and co-infiltrated in tobacco leaves for 48 h. The luciferase activity was measured according to the method in the Dual Luciferase Reporter Gene Assay Kit (11402ES60; Yeasen, Shanghai, China).

## Data Availability

All data supporting the findings of this study are available within the paper and its Supplementary Data. The GenBank accession numbers of ITS, ACT, and GAPDH for SXBJ23 are OQ119154, OQ127267, and OQ127268, respectively.

## Supplementary Materials

The following supporting information can be downloaded at: https://www.mdpi.com/2223-7747/12/10/1962/s1, Table S1: Primers used in this study.
